# 3,5-Dimeth­oxy-4′-methyl­biphen­yl

**DOI:** 10.1107/S1600536813006053

**Published:** 2013-03-09

**Authors:** Manu Lahtinen, Kalle Nättinen, Sami Nummelin

**Affiliations:** a University of Jyväskylä, Department of Chemistry, PO Box 35, FI-40014 JY, Finland; b VTT Technical Research Centre of Finland, Tampere, FIN-33101, Finland; c Molecular Materials, Department of Applied Physics, School of Science, Aalto University, PO Box 15100, FI-00076 Aalto, Finland

## Abstract

The title compound, C_15_H_16_O_2_, crystallizes with three independent mol­ecules in the asymmetric unit. The intra­molecular torsion angle between the aromatic rings of each mol­ecule are −36.4 (3), 41.3 (3) and −37.8 (3)°. In the crystal, the complicated packing of the mol­ecules forms wave-like layers along the *b* and *c* axes. The mol­ecules are connected *via* extensive meth­oxy–phenyl C—H⋯π inter­actions. A weak C—H⋯O hydrogen-bonding network also exists between meth­oxy O atoms and aromatic or meth­oxy H atoms.

## Related literature

For discussion of hydrogen bonding, see: Steiner (2002 ▶). For similar structures, see: Nakagawa *et al.* (1984 ▶); Pandi *et al.* (2000 ▶); Lahtinen *et al.* (2013*a* ▶,*b* ▶). For details of the synthesis, see: Dol *et al.* (1998 ▶); Percec *et al.* (2006 ▶). The Suzuki–Miyaura cross-coupling reaction (Miyaura & Suzuki, 1995 ▶) is widely used for the synthesis of biphenyls and related biaryl structures in organic, polymer, and supra­molecular chemistry. Such structures are frequently used as building blocks for *e.g.* precursors to liquid crystals (Solladié & Zimmermann 1984 ▶), supra­molecular polymers (Brunsveld *et al.* 2001 ▶), dendritic mol­ecules (Nummelin *et al.* 2000 ▶) as well as Percec-type self-assembling biphenyl dendrons (Percec *et al.*, 2006 ▶, 2007 ▶; Rosen *et al.*, 2009 ▶, 2010 ▶).
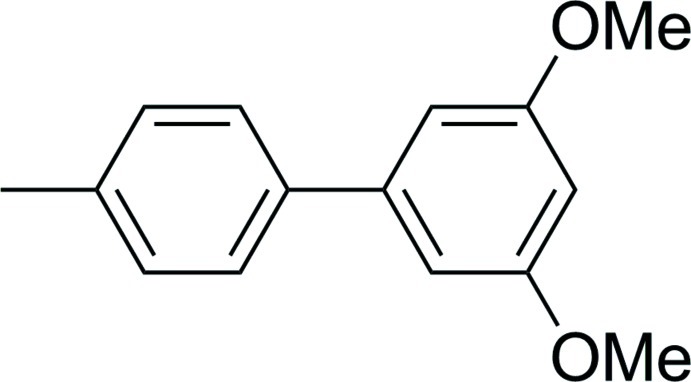


## Experimental

### 

#### Crystal data

C_15_H_16_O_2_
*M* *_r_* = 228.28
Orthorhombic, 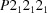

*a* = 7.16505 (18) Å
*b* = 15.3511 (4) Å
*c* = 33.3834 (8) Å
*V* = 3671.88 (16) Å^3^
*Z* = 12
Cu *K*α radiation
μ = 0.64 mm^−1^
*T* = 123 K
0.31 × 0.07 × 0.04 mm

#### Data collection

Agilent SuperNova (Dual, Cu at zero, Atlas) diffractometer
Absorption correction: analytical (*CrysAlis PRO*; Agilent, 2010 ▶) *T*_min_ = 0.900, *T*_max_ = 0.979
8494 measured reflections
5862 independent reflections
5181 reflections with *I* > 2σ(*I*)
*R*_int_ = 0.028

#### Refinement

*R*[*F* ^2^ > 2σ(*F* ^2^)] = 0.039
*wR*(*F* ^2^) = 0.094
*S* = 1.04
5862 reflections
470 parameters
H-atom parameters constrained
Δρ_max_ = 0.21 e Å^−3^
Δρ_min_ = −0.22 e Å^−3^
Absolute structure: Flack (1983 ▶), 2062 Friedel pairs
Flack parameter: 0.09 (19)

### 

Data collection: *CrysAlis PRO* (Agilent, 2010 ▶); cell refinement: *CrysAlis PRO*; data reduction: *CrysAlis PRO*; program(s) used to solve structure: *SHELXS97* (Sheldrick, 2008 ▶); program(s) used to refine structure: *SHELXL97* (Sheldrick, 2008 ▶); molecular graphics: *OLEX2* (Dolomanov *et al.*, 2009 ▶) and *Mercury* (Macrae *et al.*, 2006 ▶); software used to prepare material for publication: *OLEX2*.

## Supplementary Material

Crystal structure: contains datablock(s) I, global. DOI: 10.1107/S1600536813006053/fj2619sup1.cif

Structure factors: contains datablock(s) I. DOI: 10.1107/S1600536813006053/fj2619Isup2.hkl

Supplementary material file. DOI: 10.1107/S1600536813006053/fj2619Isup3.cml

Additional supplementary materials:  crystallographic information; 3D view; checkCIF report

## Figures and Tables

**Table 1:** Hydrogen-bond geometry (Å, °) *Cg*1–*Cg*6 are the centroids of C26–C31, C2–C7, C33–C38, C20–C25, C8–C13 and C26–C31 aromatic rings, respectively.

*D*—H⋯*A*	*D*—H	H⋯*A*	*D*⋯*A*	*D*—H⋯*A*
C29—H29⋯O15^i^	0.95	2.40	3.321 (2)	164
C25—H25⋯O51^ii^	0.95	2.84	3.459 (3)	124
C24—H24⋯O17^iii^	0.95	2.91	3.703 (3)	141
C16—H16*A*⋯O34^iv^	0.98	2.68	3.521 (3)	143
C52—H52*A*⋯O17^v^	0.98	2.63	3.398 (3)	136
C18—H18*A*⋯*Cg*1^iii^	0.98	2.89	3.686 (3)	142
C16—H16*C*⋯*Cg*2^iv^	0.98	2.62	3.366 (3)	139
C33—H33*B*⋯*Cg*3^iii^	0.98	3.05	3.476 (3)	115
C52—H52*B*⋯*Cg*4^v^	0.98	2.77	3.424 (3)	134
C35—H35*C*⋯*Cg*5^iii^	0.98	2.77	3.639 (3)	146
C35—H35*B*⋯*Cg*5	0.98	2.78	3.563 (3)	143
C18—H18*C*⋯*Cg*6^vi^	0.98	2.75	3.663 (3)	148

Symmetry codes: (i) 
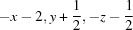
; (ii) 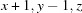; (iii) 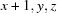; (iv) 
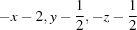
; (v) 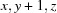; (vi) 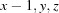.

**Fig. 1.**
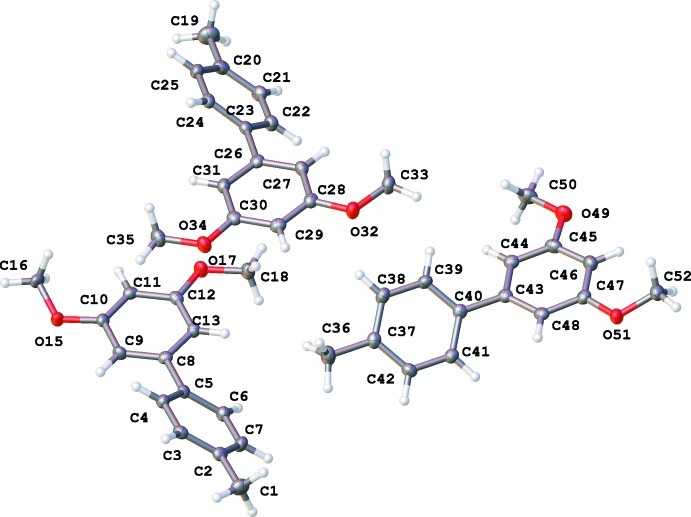
The molecular structure of the title compound showing 50% probability displacement ellipsoids and the atomic numbering.

**Fig. 2.**
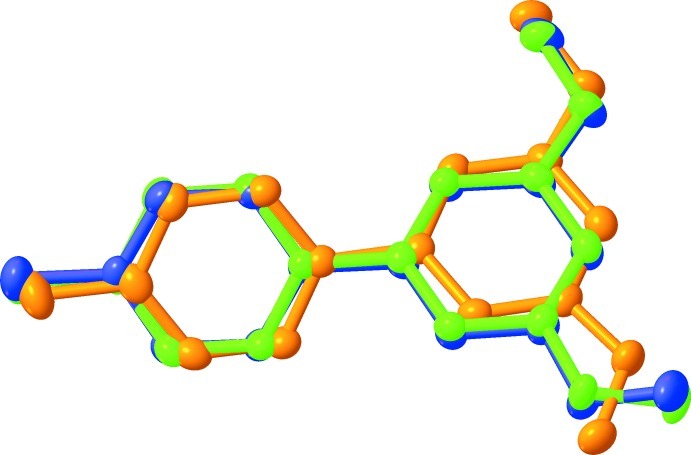
Overlay of three crystallographically disctinct molecules of an asymmetric unit.

**Fig. 3.**
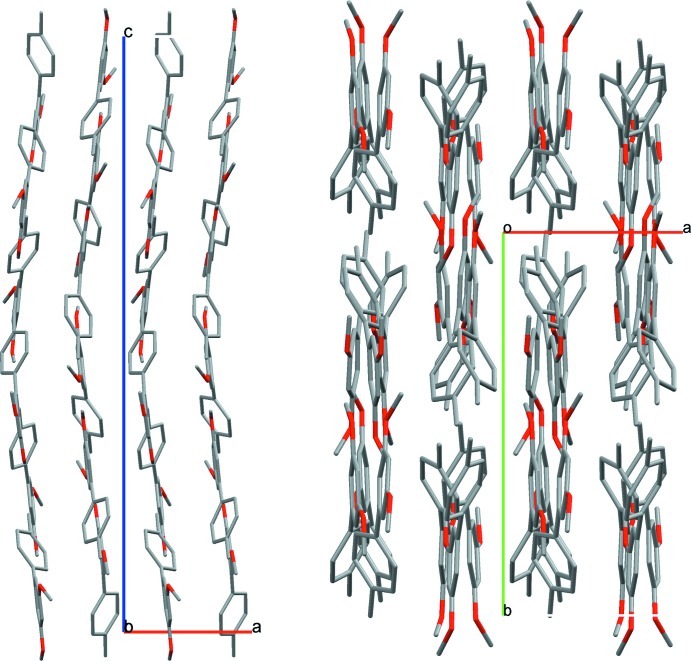
Packing of molecules along b- and c -axes, showing stacking of wave-like layers of molecules.

**Fig. 4.**
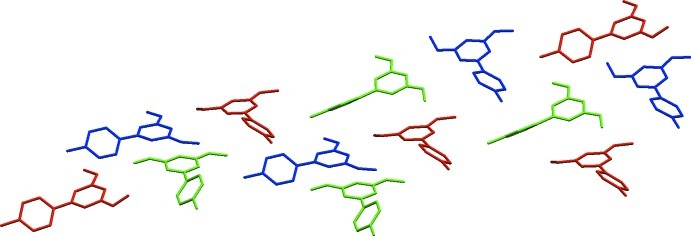
Packing order of crystallographically independent molecules on a single wave-like layer.

**Fig. 5.**
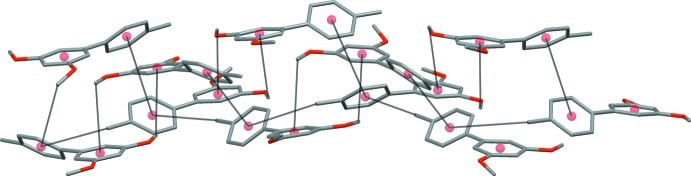
Extensive C–H···π and π–π interaction network shown along b-axis.

## supplementary crystallographic information

### Comment

The Suzuki-Miyaura cross-coupling reaction (Miyaura & Suzuki 1995) is widely used for the synthesis of biphenyls and related biaryl structures in organic, polymer, and supramolecular chemistry. Such structures are frequently used as building blocks for *e.g* precursors to liquid crystals (Solladié & Zimmermann 1984), supramolecular polymers (Brunsveld *et al.* 2001), dendritic molecules (Nummelin *et al.* 2000), and recently, Percec-type self-assembling supramolecular dendrimers (Percec *et al.* 2006, 2007; Rosen *et al.* 2009, 2010). Herein we report the crystal structure of title compound 3,5-dimethoxy-4'-methylbiphenyl (I) as a contribution to a structural study of biphenyl derivatives.

The compound (I) crystallizes in orthorhombic *P*2_1_2_1_2_1_ (No. 19) spacegroup without any solvent molecules. The asymmetric unit is consisted of three crystallographically independent but conformationally quite similar molecules (Figure 1). Major difference in conformation can be found in the orientation of one of the methoxy groups as can be seen in Figure 2. The intramolecular dihedral angles between the phenyl rings are -36.4 (3)° [C(4)–C(5)–C(8)–C(9)], 41.3 (3)° [C(22)–C(23)–C(26)–C(27)], and -37.8 (3)° [C(39)–C(40)–C(43)–C(44)], respectively. The complicated packing scheme of molecules form wave-like layers (layer on *b-* and *c*-axes) that are packed along *a*-axis (Figure 3). On each wave-like layer, molecules are orientated by 90° turns in a sequence of three crystallographically independent molecules (Figure 4). Extensive network of C–H···π and π–π interactions occur between methoxy groups and neighboring phenyl groups and between the phenyl rings (Figure 5) with distances varying from 3.366 (3) to 3.686 (3) Å and from 4.8418 (11) to 4.9137 (12) Å, respectively (Table 1). Also weak C–H···O hydrogen bond networks (Steiner 2002) exist between the methoxy groups of 3,5-dimethoxyphenyl rings and neighbouring hydrogen atoms located either on methoxy groups or in aromatic rings with D···A distances varying from 3.321 (2) to 3.703 (3) Å.

### Experimental

A flame dried Schlenk-tube was charged with 4-methylphenylboronic acid (6.00 g, 44.13 mmol), potassium fluoride (5.13 g, 88.30 mmol), 1-chloro-3,5-dimethoxybenzene (5.08 g, 29.43 mmol), Pd(OAc)_2_ (66 mg, 0.29 mmol, 1.0 mol%) and 2-(di-*tert*-butylphosphino)biphenyl (176 mg, 0.59 mmol, 2.0 mol%). The flask was sealed with a teflon screwcap, evacuated/backfilled with argon five times. Then dry, degassed THF (40 ml) was added *via* syringe. The reaction mixture was stirred at ambient temperature until the aryl chloride had been completely consumed as judged by GC analysis. The mixture was diluted with ether, filtered, and washed with 1 *M* NaOH. The aqueous layer was extracted with ether, the combined organic layer was washed with brine and dried with MgSO_4_. After evaporation the crude material was purified by flash column chromatography: silica gel/CH_2_Cl_2_. The solvent was evaporated and the product was re-crystallized from EtOH affording 6.40 g (95%) of a white crystalline solid. Crystals suitable for a single-crystal structure determination were obtained from a slow evaporation of ethanol.

### Refinement

Hydrogen atoms were calculated to their positions as riding atoms (C host) using isotropic displacement parameters that were fixed to be 1.2 or 1.5 times larger than those of the attached non-hydrogen atom.

### Crystal data

**Table:**

C_15_H_16_O_2_	*D*_x_ = 1.239 Mg m^−^^3^
*M**_r_* = 228.28	Cu *K*α radiation, λ = 1.5418 Å
Orthorhombic, *P*2_1_2_1_2_1_	Cell parameters from 4358 reflections
*a* = 7.16505 (18) Å	θ = 4.0–76.3°
*b* = 15.3511 (4) Å	µ = 0.64 mm^−^^1^
*c* = 33.3834 (8) Å	*T* = 123 K
*V* = 3671.88 (16) Å^3^	Rod, colourless
*Z* = 12	0.31 × 0.07 × 0.04 mm
*F*(000) = 1464	

### Data collection

**Table:**

Agilent SuperNova (Dual, Cu at zero, Atlas) diffractometer	5862 independent reflections
Radiation source: SuperNova (Cu) X-ray Source	5181 reflections with *I* > 2σ(*I*)
Mirror monochromator	*R*_int_ = 0.028
Detector resolution: 5.1977 pixels mm^-1^	θ_max_ = 68.0°, θ_min_ = 3.9°
ω scans	*h* = −8→8
Absorption correction: analytical (*CrysAlis PRO*; Agilent, 2010)	*k* = −12→18
*T*_min_ = 0.900, *T*_max_ = 0.979	*l* = −35→40
8494 measured reflections	

### Refinement

**Table:**

Refinement on *F*^2^	Hydrogen site location: inferred from neighbouring sites
Least-squares matrix: full	H-atom parameters constrained
*R*[*F*^2^ > 2σ(*F*^2^)] = 0.039	*w* = 1/[σ^2^(*F*_o_^2^) + (0.0416*P*)^2^] where *P* = (*F*_o_^2^ + 2*F*_c_^2^)/3
*wR*(*F*^2^) = 0.094	(Δ/σ)_max_ = 0.004
*S* = 1.04	Δρ_max_ = 0.21 e Å^−^^3^
5862 reflections	Δρ_min_ = −0.22 e Å^−^^3^
470 parameters	Extinction correction: *SHELXL97* (Sheldrick, 2008), Fc^*^=kFc[1+0.001xFc^2^λ^3^/sin(2θ)]^-1/4^
0 restraints	Extinction coefficient: 0.00022 (5)
Primary atom site location: structure-invariant direct methods	Absolute structure: Flack (1983), 2062 Friedel pairs
Secondary atom site location: difference Fourier map	Flack parameter: 0.09 (19)

### Special details

**Table:**

Geometry. All e.s.d.'s (except the e.s.d. in the dihedral angle between two l.s. planes) are estimated using the full covariance matrix. The cell e.s.d.'s are taken into account individually in the estimation of e.s.d.'s in distances, angles and torsion angles; correlations between e.s.d.'s in cell parameters are only used when they are defined by crystal symmetry. An approximate (isotropic) treatment of cell e.s.d.'s is used for estimating e.s.d.'s involving l.s. planes.
Refinement. Refinement of *F*^2^ against ALL reflections. The weighted *R*-factor *wR* and goodness of fit *S* are based on *F*^2^, conventional *R*-factors *R* are based on *F*, with *F* set to zero for negative *F*^2^. The threshold expression of *F*^2^ > σ(*F*^2^) is used only for calculating *R*-factors(gt) *etc*. and is not relevant to the choice of reflections for refinement. *R*-factors based on *F*^2^ are statistically about twice as large as those based on *F*, and *R*- factors based on ALL data will be even larger.

### Fractional atomic coordinates and isotropic or equivalent isotropic displacement parameters (Å^2^)

**Table:**

	*x*	*y*	*z*	*U*_iso_*/*U*_eq_	
O32	−0.6581 (3)	0.00455 (9)	−0.13539 (4)	0.0257 (3)	
O15	−1.2140 (2)	−0.53532 (9)	−0.24070 (4)	0.0230 (3)	
O17	−1.1815 (3)	−0.31557 (9)	−0.14009 (4)	0.0256 (3)	
O34	−0.7204 (2)	−0.24734 (10)	−0.21097 (4)	0.0257 (3)	
O51	−1.1549 (3)	0.51057 (9)	−0.07803 (4)	0.0258 (3)	
C48	−1.1888 (3)	0.36199 (13)	−0.09024 (6)	0.0203 (4)	
H48	−1.2070	0.3761	−0.1177	0.024*	
C31	−0.6807 (3)	−0.26527 (13)	−0.13890 (6)	0.0195 (4)	
H31	−0.6843	−0.3270	−0.1407	0.023*	
O49	−1.1295 (3)	0.30941 (10)	0.03053 (4)	0.0287 (4)	
C8	−1.2439 (3)	−0.30112 (13)	−0.24971 (6)	0.0179 (4)	
C11	−1.1978 (3)	−0.42464 (13)	−0.18851 (6)	0.0188 (4)	
H11	−1.1833	−0.4665	−0.1678	0.023*	
C46	−1.1387 (3)	0.40899 (14)	−0.02203 (6)	0.0233 (4)	
H46	−1.1189	0.4541	−0.0030	0.028*	
C44	−1.1719 (3)	0.25491 (14)	−0.03740 (6)	0.0206 (4)	
H44	−1.1763	0.1960	−0.0288	0.025*	
C13	−1.2266 (3)	−0.27383 (13)	−0.20985 (6)	0.0187 (4)	
H13	−1.2312	−0.2136	−0.2035	0.022*	
C23	−0.6510 (3)	−0.27891 (13)	−0.06500 (6)	0.0196 (4)	
C45	−1.1468 (3)	0.32131 (15)	−0.00984 (6)	0.0224 (4)	
C35	−0.7262 (4)	−0.34030 (14)	−0.21533 (6)	0.0276 (5)	
H35A	−0.7526	−0.3551	−0.2433	0.041*	
H35B	−0.8245	−0.3642	−0.1981	0.041*	
H35C	−0.6055	−0.3651	−0.2075	0.041*	
C10	−1.2146 (3)	−0.45040 (13)	−0.22815 (6)	0.0191 (4)	
C30	−0.6954 (3)	−0.21431 (14)	−0.17353 (6)	0.0207 (4)	
C28	−0.6658 (3)	−0.08472 (13)	−0.13408 (6)	0.0211 (4)	
C36	−1.2425 (4)	−0.00048 (14)	−0.19728 (7)	0.0307 (5)	
H36A	−1.1237	−0.0051	−0.2117	0.046*	
H36B	−1.3426	0.0127	−0.2163	0.046*	
H36C	−1.2694	−0.0558	−0.1838	0.046*	
C2	−1.2986 (3)	−0.10882 (13)	−0.34317 (6)	0.0221 (4)	
C5	−1.2669 (3)	−0.23535 (12)	−0.28198 (6)	0.0174 (4)	
C43	−1.1908 (3)	0.27539 (14)	−0.07817 (6)	0.0201 (4)	
C26	−0.6609 (3)	−0.22480 (13)	−0.10173 (6)	0.0200 (4)	
C27	−0.6522 (3)	−0.13359 (13)	−0.09919 (6)	0.0203 (4)	
H27	−0.6371	−0.1057	−0.0740	0.024*	
C9	−1.2396 (3)	−0.38929 (13)	−0.25875 (6)	0.0197 (4)	
H9	−1.2536	−0.4081	−0.2857	0.024*	
C38	−1.1136 (3)	0.06328 (14)	−0.13330 (6)	0.0239 (5)	
H38	−1.0395	0.0124	−0.1303	0.029*	
C29	−0.6881 (3)	−0.12423 (14)	−0.17108 (6)	0.0223 (4)	
H29	−0.6983	−0.0898	−0.1946	0.027*	
C39	−1.1042 (3)	0.12794 (14)	−0.10462 (6)	0.0208 (4)	
H39	−1.0263	0.1200	−0.0819	0.025*	
C21	−0.7427 (3)	−0.30780 (14)	0.00358 (6)	0.0235 (4)	
H21	−0.8070	−0.2896	0.0270	0.028*	
C6	−1.3671 (3)	−0.15842 (13)	−0.27573 (6)	0.0201 (4)	
H6	−1.4261	−0.1485	−0.2507	0.024*	
C3	−1.2022 (3)	−0.18594 (13)	−0.34947 (6)	0.0206 (4)	
H3	−1.1473	−0.1965	−0.3749	0.025*	
C25	−0.5491 (3)	−0.40961 (14)	−0.03083 (7)	0.0236 (5)	
H25	−0.4799	−0.4624	−0.0312	0.028*	
C24	−0.5494 (3)	−0.35747 (13)	−0.06463 (6)	0.0204 (4)	
H24	−0.4809	−0.3747	−0.0877	0.025*	
C40	−1.2074 (3)	0.20496 (13)	−0.10848 (6)	0.0194 (4)	
C4	−1.1841 (3)	−0.24814 (13)	−0.31951 (6)	0.0197 (4)	
H4	−1.1148	−0.2998	−0.3245	0.024*	
C50	−1.1437 (4)	0.22174 (15)	0.04516 (6)	0.0280 (5)	
H50A	−1.0489	0.1854	0.0321	0.042*	
H50B	−1.2681	0.1987	0.0391	0.042*	
H50C	−1.1237	0.2212	0.0742	0.042*	
C20	−0.6471 (3)	−0.38717 (14)	0.00367 (6)	0.0246 (4)	
C41	−1.3212 (3)	0.21389 (13)	−0.14206 (6)	0.0207 (4)	
H41	−1.3919	0.2657	−0.1455	0.025*	
C22	−0.7454 (3)	−0.25507 (14)	−0.03023 (6)	0.0236 (5)	
H22	−0.8129	−0.2018	−0.0296	0.028*	
C47	−1.1602 (3)	0.42827 (14)	−0.06234 (6)	0.0214 (4)	
C18	−1.1755 (4)	−0.22542 (13)	−0.12935 (6)	0.0248 (5)	
H18A	−1.0726	−0.1969	−0.1436	0.037*	
H18B	−1.1563	−0.2200	−0.1004	0.037*	
H18C	−1.2937	−0.1976	−0.1368	0.037*	
C12	−1.2028 (3)	−0.33505 (13)	−0.17976 (6)	0.0193 (4)	
C37	−1.2300 (3)	0.07136 (13)	−0.16657 (6)	0.0212 (4)	
C42	−1.3328 (3)	0.14800 (14)	−0.17063 (6)	0.0227 (4)	
H42	−1.4120	0.1554	−0.1932	0.027*	
C16	−1.1429 (3)	−0.59988 (13)	−0.21377 (6)	0.0249 (5)	
H16A	−1.1382	−0.6564	−0.2274	0.037*	
H16B	−1.2248	−0.6041	−0.1903	0.037*	
H16C	−1.0170	−0.5834	−0.2051	0.037*	
C33	−0.6269 (4)	0.05009 (13)	−0.09882 (7)	0.0270 (5)	
H33A	−0.6359	0.1129	−0.1036	0.041*	
H33B	−0.5023	0.0360	−0.0886	0.041*	
H33C	−0.7211	0.0326	−0.0791	0.041*	
C7	−1.3810 (3)	−0.09623 (14)	−0.30590 (7)	0.0236 (5)	
H7	−1.4483	−0.0440	−0.3009	0.028*	
C52	−1.0702 (4)	0.57706 (15)	−0.05435 (7)	0.0284 (5)	
H52A	−1.0571	0.6302	−0.0704	0.043*	
H52B	−0.9467	0.5576	−0.0455	0.043*	
H52C	−1.1485	0.5891	−0.0309	0.043*	
C1	−1.3141 (4)	−0.04042 (15)	−0.37556 (7)	0.0313 (5)	
H1A	−1.2136	0.0023	−0.3724	0.047*	
H1B	−1.4350	−0.0110	−0.3734	0.047*	
H1C	−1.3036	−0.0682	−0.4019	0.047*	
C19	−0.6454 (4)	−0.44453 (18)	0.04011 (7)	0.0378 (6)	
H19A	−0.5222	−0.4419	0.0528	0.057*	
H19B	−0.6724	−0.5047	0.0322	0.057*	
H19C	−0.7405	−0.4244	0.0591	0.057*	

### Atomic displacement parameters (Å^2^)

**Table:**

	*U*^11^	*U*^22^	*U*^33^	*U*^12^	*U*^13^	*U*^23^
O32	0.0337 (9)	0.0178 (7)	0.0256 (7)	−0.0004 (7)	−0.0006 (7)	−0.0008 (6)
O15	0.0326 (9)	0.0153 (6)	0.0210 (7)	0.0011 (7)	−0.0057 (6)	0.0012 (5)
O17	0.0373 (9)	0.0223 (7)	0.0172 (6)	0.0002 (7)	−0.0014 (7)	−0.0015 (6)
O34	0.0337 (9)	0.0237 (7)	0.0197 (7)	−0.0028 (7)	−0.0015 (7)	−0.0025 (6)
O51	0.0322 (9)	0.0180 (7)	0.0273 (7)	−0.0013 (7)	−0.0057 (7)	−0.0014 (6)
C48	0.0191 (10)	0.0235 (10)	0.0185 (9)	0.0014 (9)	0.0003 (8)	−0.0002 (8)
C31	0.0174 (10)	0.0167 (8)	0.0244 (9)	0.0024 (8)	0.0015 (8)	−0.0010 (8)
O49	0.0370 (10)	0.0308 (8)	0.0184 (7)	0.0024 (8)	−0.0023 (7)	−0.0006 (6)
C8	0.0128 (9)	0.0209 (9)	0.0200 (9)	0.0006 (8)	0.0017 (8)	0.0015 (8)
C11	0.0159 (10)	0.0195 (9)	0.0210 (9)	−0.0010 (8)	−0.0006 (8)	0.0039 (8)
C46	0.0215 (11)	0.0249 (10)	0.0234 (10)	0.0008 (9)	−0.0015 (9)	−0.0046 (8)
C44	0.0179 (10)	0.0234 (10)	0.0204 (10)	−0.0006 (8)	0.0013 (8)	0.0024 (8)
C13	0.0187 (10)	0.0164 (9)	0.0211 (9)	−0.0010 (8)	0.0019 (8)	−0.0009 (8)
C23	0.0171 (10)	0.0193 (9)	0.0224 (9)	−0.0008 (9)	0.0000 (8)	−0.0021 (8)
C45	0.0179 (10)	0.0319 (11)	0.0174 (9)	−0.0003 (9)	−0.0002 (8)	−0.0013 (9)
C35	0.0298 (12)	0.0283 (11)	0.0246 (10)	−0.0031 (10)	0.0030 (9)	−0.0075 (9)
C10	0.0162 (10)	0.0165 (9)	0.0246 (10)	−0.0004 (8)	−0.0019 (8)	0.0003 (8)
C30	0.0174 (10)	0.0249 (10)	0.0196 (9)	0.0004 (9)	0.0014 (8)	−0.0017 (8)
C28	0.0162 (10)	0.0196 (9)	0.0276 (10)	0.0003 (8)	0.0017 (9)	−0.0001 (8)
C36	0.0411 (15)	0.0238 (10)	0.0272 (11)	−0.0018 (11)	0.0023 (10)	−0.0035 (9)
C2	0.0215 (11)	0.0207 (10)	0.0240 (9)	−0.0045 (9)	−0.0022 (9)	0.0031 (8)
C5	0.0169 (10)	0.0155 (8)	0.0200 (9)	−0.0021 (8)	−0.0011 (8)	−0.0003 (8)
C43	0.0173 (9)	0.0238 (10)	0.0193 (9)	0.0014 (9)	0.0013 (8)	−0.0002 (8)
C26	0.0163 (10)	0.0214 (9)	0.0223 (9)	0.0017 (9)	0.0020 (8)	−0.0003 (8)
C27	0.0199 (10)	0.0216 (10)	0.0194 (9)	−0.0016 (9)	0.0014 (8)	−0.0032 (8)
C9	0.0227 (10)	0.0214 (9)	0.0148 (8)	−0.0005 (9)	−0.0010 (8)	−0.0006 (8)
C38	0.0263 (11)	0.0183 (9)	0.0271 (11)	0.0018 (9)	0.0039 (9)	0.0021 (9)
C29	0.0193 (10)	0.0253 (10)	0.0223 (9)	0.0025 (9)	0.0015 (9)	0.0024 (8)
C39	0.0217 (11)	0.0215 (10)	0.0192 (9)	0.0014 (9)	−0.0008 (8)	0.0031 (8)
C21	0.0234 (11)	0.0271 (10)	0.0199 (9)	0.0019 (9)	0.0026 (8)	−0.0035 (8)
C6	0.0198 (10)	0.0198 (9)	0.0206 (9)	−0.0005 (9)	0.0017 (8)	−0.0009 (8)
C3	0.0214 (11)	0.0237 (10)	0.0168 (9)	−0.0026 (9)	−0.0009 (8)	0.0006 (8)
C25	0.0248 (11)	0.0194 (10)	0.0267 (11)	0.0021 (9)	−0.0041 (9)	−0.0018 (9)
C24	0.0197 (10)	0.0192 (10)	0.0224 (10)	−0.0006 (9)	0.0012 (8)	−0.0035 (8)
C40	0.0209 (10)	0.0186 (9)	0.0188 (9)	−0.0012 (8)	0.0043 (8)	0.0025 (8)
C4	0.0206 (10)	0.0182 (9)	0.0203 (9)	0.0018 (8)	−0.0011 (8)	−0.0005 (7)
C50	0.0285 (12)	0.0354 (12)	0.0202 (9)	−0.0014 (11)	−0.0021 (9)	0.0042 (9)
C20	0.0233 (11)	0.0262 (10)	0.0243 (10)	−0.0050 (10)	−0.0044 (9)	0.0027 (9)
C41	0.0214 (10)	0.0185 (9)	0.0223 (9)	0.0001 (8)	0.0025 (8)	0.0013 (8)
C22	0.0262 (12)	0.0224 (10)	0.0223 (10)	0.0028 (9)	0.0003 (9)	−0.0025 (8)
C47	0.0171 (10)	0.0211 (9)	0.0261 (10)	0.0015 (9)	0.0016 (9)	0.0004 (8)
C18	0.0307 (12)	0.0222 (10)	0.0214 (9)	0.0003 (10)	0.0015 (9)	−0.0042 (8)
C12	0.0159 (9)	0.0235 (10)	0.0186 (9)	−0.0034 (8)	0.0007 (8)	−0.0017 (8)
C37	0.0217 (11)	0.0195 (9)	0.0223 (10)	−0.0034 (9)	0.0043 (8)	0.0006 (8)
C42	0.0236 (11)	0.0266 (10)	0.0178 (9)	−0.0034 (9)	−0.0012 (9)	−0.0002 (8)
C16	0.0279 (12)	0.0158 (9)	0.0311 (11)	0.0018 (9)	−0.0089 (10)	0.0044 (8)
C33	0.0321 (13)	0.0186 (9)	0.0304 (11)	−0.0005 (9)	0.0027 (10)	−0.0024 (9)
C7	0.0230 (11)	0.0188 (9)	0.0292 (11)	0.0045 (9)	0.0002 (9)	−0.0001 (9)
C52	0.0297 (12)	0.0204 (10)	0.0351 (12)	−0.0008 (10)	−0.0064 (10)	−0.0031 (9)
C1	0.0345 (13)	0.0281 (11)	0.0313 (11)	−0.0005 (11)	−0.0011 (10)	0.0086 (10)
C19	0.0400 (15)	0.0429 (14)	0.0306 (12)	0.0002 (13)	0.0005 (12)	0.0098 (11)

### Geometric parameters (Å, º)

**Table:**

O32—C28	1.372 (3)	C43—C40	1.486 (3)
O32—C33	1.424 (3)	C26—C27	1.404 (3)
O15—C10	1.369 (2)	C27—H27	0.9500
O15—C16	1.432 (2)	C9—H9	0.9500
O17—C18	1.430 (2)	C38—H38	0.9500
O17—C12	1.366 (2)	C38—C39	1.381 (3)
O34—C35	1.435 (3)	C38—C37	1.394 (3)
O34—C30	1.361 (3)	C29—H29	0.9500
O51—C47	1.368 (3)	C39—H39	0.9500
O51—C52	1.427 (3)	C39—C40	1.400 (3)
C48—H48	0.9500	C21—H21	0.9500
C48—C43	1.389 (3)	C21—C20	1.398 (3)
C48—C47	1.394 (3)	C21—C22	1.389 (3)
C31—H31	0.9500	C6—H6	0.9500
C31—C30	1.400 (3)	C6—C7	1.391 (3)
C31—C26	1.395 (3)	C3—H3	0.9500
O49—C45	1.366 (3)	C3—C4	1.389 (3)
O49—C50	1.435 (3)	C25—H25	0.9500
C8—C13	1.401 (3)	C25—C24	1.383 (3)
C8—C5	1.486 (3)	C25—C20	1.392 (3)
C8—C9	1.387 (3)	C24—H24	0.9500
C11—H11	0.9500	C40—C41	1.393 (3)
C11—C10	1.386 (3)	C4—H4	0.9500
C11—C12	1.406 (3)	C50—H50A	0.9800
C46—H46	0.9500	C50—H50B	0.9800
C46—C45	1.407 (3)	C50—H50C	0.9800
C46—C47	1.387 (3)	C20—C19	1.502 (3)
C44—H44	0.9500	C41—H41	0.9500
C44—C45	1.385 (3)	C41—C42	1.393 (3)
C44—C43	1.403 (3)	C22—H22	0.9500
C13—H13	0.9500	C18—H18A	0.9800
C13—C12	1.386 (3)	C18—H18B	0.9800
C23—C26	1.483 (3)	C18—H18C	0.9800
C23—C24	1.409 (3)	C37—C42	1.395 (3)
C23—C22	1.392 (3)	C42—H42	0.9500
C35—H35A	0.9800	C16—H16A	0.9800
C35—H35B	0.9800	C16—H16B	0.9800
C35—H35C	0.9800	C16—H16C	0.9800
C10—C9	1.398 (3)	C33—H33A	0.9800
C30—C29	1.386 (3)	C33—H33B	0.9800
C28—C27	1.389 (3)	C33—H33C	0.9800
C28—C29	1.385 (3)	C7—H7	0.9500
C36—H36A	0.9800	C52—H52A	0.9800
C36—H36B	0.9800	C52—H52B	0.9800
C36—H36C	0.9800	C52—H52C	0.9800
C36—C37	1.508 (3)	C1—H1A	0.9800
C2—C3	1.386 (3)	C1—H1B	0.9800
C2—C7	1.391 (3)	C1—H1C	0.9800
C2—C1	1.511 (3)	C19—H19A	0.9800
C5—C6	1.397 (3)	C19—H19B	0.9800
C5—C4	1.400 (3)	C19—H19C	0.9800
			
C28—O32—C33	117.98 (16)	C5—C6—H6	119.7
C10—O15—C16	117.92 (16)	C7—C6—C5	120.57 (19)
C12—O17—C18	117.26 (16)	C7—C6—H6	119.7
C30—O34—C35	117.86 (17)	C2—C3—H3	119.2
C47—O51—C52	117.42 (17)	C2—C3—C4	121.64 (19)
C43—C48—H48	119.8	C4—C3—H3	119.2
C43—C48—C47	120.40 (18)	C24—C25—H25	119.0
C47—C48—H48	119.8	C24—C25—C20	122.1 (2)
C30—C31—H31	120.2	C20—C25—H25	119.0
C26—C31—H31	120.2	C23—C24—H24	119.9
C26—C31—C30	119.57 (18)	C25—C24—C23	120.2 (2)
C45—O49—C50	117.04 (17)	C25—C24—H24	119.9
C13—C8—C5	119.70 (18)	C39—C40—C43	120.62 (19)
C9—C8—C13	119.78 (18)	C41—C40—C43	121.57 (19)
C9—C8—C5	120.53 (18)	C41—C40—C39	117.79 (18)
C10—C11—H11	120.8	C5—C4—H4	119.7
C10—C11—C12	118.35 (18)	C3—C4—C5	120.54 (19)
C12—C11—H11	120.8	C3—C4—H4	119.7
C45—C46—H46	120.7	O49—C50—H50A	109.5
C47—C46—H46	120.7	O49—C50—H50B	109.5
C47—C46—C45	118.7 (2)	O49—C50—H50C	109.5
C45—C44—H44	120.3	H50A—C50—H50B	109.5
C45—C44—C43	119.47 (19)	H50A—C50—H50C	109.5
C43—C44—H44	120.3	H50B—C50—H50C	109.5
C8—C13—H13	120.1	C21—C20—C19	121.1 (2)
C12—C13—C8	119.77 (18)	C25—C20—C21	117.45 (19)
C12—C13—H13	120.1	C25—C20—C19	121.4 (2)
C24—C23—C26	120.77 (18)	C40—C41—H41	119.5
C22—C23—C26	121.26 (19)	C42—C41—C40	120.98 (19)
C22—C23—C24	117.94 (19)	C42—C41—H41	119.5
O49—C45—C46	114.17 (19)	C23—C22—H22	119.4
O49—C45—C44	124.7 (2)	C21—C22—C23	121.2 (2)
C44—C45—C46	121.15 (19)	C21—C22—H22	119.4
O34—C35—H35A	109.5	O51—C47—C48	114.98 (18)
O34—C35—H35B	109.5	O51—C47—C46	124.4 (2)
O34—C35—H35C	109.5	C46—C47—C48	120.6 (2)
H35A—C35—H35B	109.5	O17—C18—H18A	109.5
H35A—C35—H35C	109.5	O17—C18—H18B	109.5
H35B—C35—H35C	109.5	O17—C18—H18C	109.5
O15—C10—C11	124.29 (18)	H18A—C18—H18B	109.5
O15—C10—C9	114.56 (18)	H18A—C18—H18C	109.5
C11—C10—C9	121.13 (18)	H18B—C18—H18C	109.5
O34—C30—C31	124.08 (19)	O17—C12—C11	114.36 (18)
O34—C30—C29	115.53 (19)	O17—C12—C13	124.59 (18)
C29—C30—C31	120.4 (2)	C13—C12—C11	121.05 (18)
O32—C28—C27	124.28 (19)	C38—C37—C36	120.8 (2)
O32—C28—C29	114.43 (19)	C38—C37—C42	117.93 (19)
C29—C28—C27	121.29 (18)	C42—C37—C36	121.3 (2)
H36A—C36—H36B	109.5	C41—C42—C37	121.0 (2)
H36A—C36—H36C	109.5	C41—C42—H42	119.5
H36B—C36—H36C	109.5	C37—C42—H42	119.5
C37—C36—H36A	109.5	O15—C16—H16A	109.5
C37—C36—H36B	109.5	O15—C16—H16B	109.5
C37—C36—H36C	109.5	O15—C16—H16C	109.5
C3—C2—C7	117.76 (19)	H16A—C16—H16B	109.5
C3—C2—C1	121.4 (2)	H16A—C16—H16C	109.5
C7—C2—C1	120.8 (2)	H16B—C16—H16C	109.5
C6—C5—C8	121.55 (18)	O32—C33—H33A	109.5
C6—C5—C4	118.02 (18)	O32—C33—H33B	109.5
C4—C5—C8	120.41 (18)	O32—C33—H33C	109.5
C48—C43—C44	119.65 (19)	H33A—C33—H33B	109.5
C48—C43—C40	119.98 (18)	H33A—C33—H33C	109.5
C44—C43—C40	120.35 (19)	H33B—C33—H33C	109.5
C31—C26—C23	119.41 (18)	C2—C7—C6	121.5 (2)
C31—C26—C27	120.16 (19)	C2—C7—H7	119.3
C27—C26—C23	120.43 (18)	C6—C7—H7	119.3
C28—C27—C26	119.00 (19)	O51—C52—H52A	109.5
C28—C27—H27	120.5	O51—C52—H52B	109.5
C26—C27—H27	120.5	O51—C52—H52C	109.5
C8—C9—C10	119.91 (18)	H52A—C52—H52B	109.5
C8—C9—H9	120.0	H52A—C52—H52C	109.5
C10—C9—H9	120.0	H52B—C52—H52C	109.5
C39—C38—H38	119.4	C2—C1—H1A	109.5
C39—C38—C37	121.2 (2)	C2—C1—H1B	109.5
C37—C38—H38	119.4	C2—C1—H1C	109.5
C30—C29—H29	120.2	H1A—C1—H1B	109.5
C28—C29—C30	119.6 (2)	H1A—C1—H1C	109.5
C28—C29—H29	120.2	H1B—C1—H1C	109.5
C38—C39—H39	119.4	C20—C19—H19A	109.5
C38—C39—C40	121.1 (2)	C20—C19—H19B	109.5
C40—C39—H39	119.4	C20—C19—H19C	109.5
C20—C21—H21	119.4	H19A—C19—H19B	109.5
C22—C21—H21	119.4	H19A—C19—H19C	109.5
C22—C21—C20	121.1 (2)	H19B—C19—H19C	109.5
			
O32—C28—C27—C26	−179.7 (2)	C9—C8—C5—C6	145.2 (2)
O32—C28—C29—C30	179.2 (2)	C9—C8—C5—C4	−36.4 (3)
O15—C10—C9—C8	179.6 (2)	C38—C39—C40—C43	−177.8 (2)
O34—C30—C29—C28	179.04 (19)	C38—C39—C40—C41	0.5 (3)
C48—C43—C40—C39	140.4 (2)	C38—C37—C42—C41	−0.7 (3)
C48—C43—C40—C41	−37.8 (3)	C29—C28—C27—C26	0.1 (3)
C31—C30—C29—C28	0.1 (4)	C39—C38—C37—C36	−178.6 (2)
C31—C26—C27—C28	0.8 (3)	C39—C38—C37—C42	1.8 (3)
C8—C13—C12—O17	178.9 (2)	C39—C40—C41—C42	0.5 (3)
C8—C13—C12—C11	−0.8 (3)	C6—C5—C4—C3	−0.2 (3)
C8—C5—C6—C7	177.6 (2)	C3—C2—C7—C6	0.3 (3)
C8—C5—C4—C3	−178.69 (19)	C24—C23—C26—C31	40.2 (3)
C11—C10—C9—C8	1.4 (3)	C24—C23—C26—C27	−140.4 (2)
C44—C43—C40—C39	−37.8 (3)	C24—C23—C22—C21	−1.2 (3)
C44—C43—C40—C41	144.0 (2)	C24—C25—C20—C21	−1.9 (3)
C13—C8—C5—C6	−34.7 (3)	C24—C25—C20—C19	180.0 (2)
C13—C8—C5—C4	143.7 (2)	C40—C41—C42—C37	−0.4 (3)
C13—C8—C9—C10	−1.2 (3)	C4—C5—C6—C7	−0.9 (3)
C23—C26—C27—C28	−178.7 (2)	C50—O49—C45—C46	177.7 (2)
C45—C46—C47—O51	−179.9 (2)	C50—O49—C45—C44	−1.8 (3)
C45—C46—C47—C48	−0.2 (4)	C20—C21—C22—C23	−0.8 (4)
C45—C44—C43—C48	−1.6 (3)	C20—C25—C24—C23	−0.1 (3)
C45—C44—C43—C40	176.6 (2)	C22—C23—C26—C31	−138.1 (2)
C35—O34—C30—C31	−2.1 (3)	C22—C23—C26—C27	41.3 (3)
C35—O34—C30—C29	179.1 (2)	C22—C23—C24—C25	1.7 (3)
C10—C11—C12—O17	−178.73 (18)	C22—C21—C20—C25	2.4 (3)
C10—C11—C12—C13	0.9 (3)	C22—C21—C20—C19	−179.5 (2)
C30—C31—C26—C23	178.2 (2)	C47—C48—C43—C44	2.7 (3)
C30—C31—C26—C27	−1.2 (3)	C47—C48—C43—C40	−175.5 (2)
C36—C37—C42—C41	179.7 (2)	C47—C46—C45—O49	−178.2 (2)
C2—C3—C4—C5	1.4 (3)	C47—C46—C45—C44	1.3 (4)
C5—C8—C13—C12	−179.2 (2)	C18—O17—C12—C11	176.9 (2)
C5—C8—C9—C10	178.9 (2)	C18—O17—C12—C13	−2.8 (3)
C5—C6—C7—C2	0.8 (3)	C12—C11—C10—O15	−179.3 (2)
C43—C48—C47—O51	177.9 (2)	C12—C11—C10—C9	−1.2 (3)
C43—C48—C47—C46	−1.8 (3)	C37—C38—C39—C40	−1.7 (3)
C43—C44—C45—O49	179.0 (2)	C16—O15—C10—C11	−17.5 (3)
C43—C44—C45—C46	−0.4 (3)	C16—O15—C10—C9	164.4 (2)
C43—C40—C41—C42	178.8 (2)	C33—O32—C28—C27	2.6 (3)
C26—C31—C30—O34	−178.0 (2)	C33—O32—C28—C29	−177.2 (2)
C26—C31—C30—C29	0.8 (3)	C7—C2—C3—C4	−1.4 (3)
C26—C23—C24—C25	−176.7 (2)	C52—O51—C47—C48	−160.9 (2)
C26—C23—C22—C21	177.1 (2)	C52—O51—C47—C46	18.8 (3)
C27—C28—C29—C30	−0.6 (3)	C1—C2—C3—C4	178.5 (2)
C9—C8—C13—C12	0.9 (3)	C1—C2—C7—C6	−179.7 (2)

### Hydrogen-bond geometry (Å, º)

Cg1–Cg6 are the centroids of C26–C31, C2–C7, C33–C38, C20–C25, C8–C13 and C26–C31 aromatic rings, respectively.

**Table:**

*D*—H···*A*	*D*—H	H···*A*	*D*···*A*	*D*—H···*A*
C29—H29···O15^i^	0.95	2.40	3.321 (2)	164
C25—H25···O51^ii^	0.95	2.84	3.459 (3)	124
C24—H24···O17^iii^	0.95	2.91	3.703 (3)	141
C16—H16*A*···O34^iv^	0.98	2.68	3.521 (3)	143
C52—H52*A*···O17^v^	0.98	2.63	3.398 (3)	136
C18—H18*A*···*Cg*1^iii^	0.98	2.89	3.686 (3)	142
C16—H16*C*···*Cg*2^iv^	0.98	2.62	3.366 (3)	139
C33—H33*B*···*Cg*3^iii^	0.98	3.05	3.476 (3)	115
C52—H52*B*···*Cg*4^v^	0.98	2.77	3.424 (3)	134
C35—H35*C*···*Cg*5^iii^	0.98	2.77	3.639 (3)	146
C35—H35*B*···*Cg*5	0.98	2.78	3.563 (3)	143
C18—H18*C*···*Cg*6^vi^	0.98	2.75	3.663 (3)	148

Symmetry codes: (i) −*x*−2, *y*+1/2, −*z*−1/2; (ii) *x*+1, *y*−1, *z*; (iii) *x*+1, *y*, *z*; (iv) −*x*−2, *y*−1/2, −*z*−1/2; (v) *x*, *y*+1, *z*; (vi) *x*−1, *y*, *z*.
